# Caseous calcification of the mitral annulus as a rare cause of embolic stroke in a young patient with end-stage renal disease: a case report

**DOI:** 10.1093/ehjcr/ytag159

**Published:** 2026-03-06

**Authors:** Faten Yahia, Emna Jarrar, Amira Ben Afia, Sana Ben Amor, Elyes Neffati

**Affiliations:** Department of Cardiology, Sahloul University Hospital, Route de ceinture, 4054 Sahloul, Sousse, Tunisia; Department of Neurology, Sahloul University Hospital, Route de ceinture, 4054 Sahloul, Sousse, Tunisia; Department of Radiology, Sahloul University Hospital, Route de ceinture, 4054 Sahloul, Sousse, Tunisia; Department of Neurology, Sahloul University Hospital, Route de ceinture, 4054 Sahloul, Sousse, Tunisia; Department of Cardiology, Sahloul University Hospital, Route de ceinture, 4054 Sahloul, Sousse, Tunisia

**Keywords:** Caseous mitral annular calcification, Embolic stroke, Echocardiography, End-stage renal disease, Multimodality imaging, Case report

## Abstract

**Background:**

Caseous calcification of the mitral annulus (CCMA) is a rare form of mitral annular calcification and may be an embolic source, particularly in end-stage renal disease (ESRD).

**Case summary:**

A 33-year-old man with ESRD on haemodialysis presented with acute left middle cerebral artery stroke (NIHSS 21). Brain CT angiography showed a dense calcified embolus occluding the proximal M1 segment. Echocardiography and ECG-gated non-contrast cardiac CT identified two posterior annular lesions with a calcified rim and central low-attenuation, consistent with CCMA. The close radiological match between the intracranial calcified embolus and the annular lesions suggested CCMA as the most plausible source. Surgical excision was deferred after multidisciplinary discussion because of the high haemorrhagic risk related to the large recent infarction. The patient was managed conservatively with oral anticoagulation and strict blood pressure control.

**Discussion:**

This case illustrates that CCMA may be a possible embolic source even in young dialysis patients. It underscores the diagnostic value of multimodality imaging and the importance of individualized multidisciplinary decision-making when weighing embolic and haemorrhagic risks.

Learning points

- Caseous calcification of the mitral annulus, though rare, should be considered as a potential embolic source in young patients with end-stage renal disease presenting with stroke.
- Multimodality imaging is essential to differentiate CCMA from other cardiac masses and to guide management decisions.
- Individualized multidisciplinary discussion is key to balancing surgical risk and anticoagulation strategies in patients with CCMA.

## Introduction

Mitral annular calcification (MAC) represents a chronic degenerative process of the mitral valve fibrous ring, most often observed in elderly patients, women, and those with end-stage renal disease (ESRD) on dialysis.^1^ Caseous calcification of the mitral annulus (CCMA) is an uncommon MAC variant characterized by liquefaction necrosis within calcified deposits, with an estimated prevalence of around 0.06–0.07% in echocardiographic series and represents about 0.6% of MAC cases.^2^

Several reports suggest that CCMA may carry a higher risk of systemic embolization than conventional MAC, particularly cerebrovascular events in patients with chronic kidney disease.^3,4^ Although most reported patients are elderly with multiple comorbidities or advanced renal disease, similar presentations have been described in younger adults on dialysis.^5^ The present case adds to this limited literature and highlights the diagnostic and therapeutic considerations of CCMA in a young patient with ESRD.

## Case presentation

**Figure 1:**
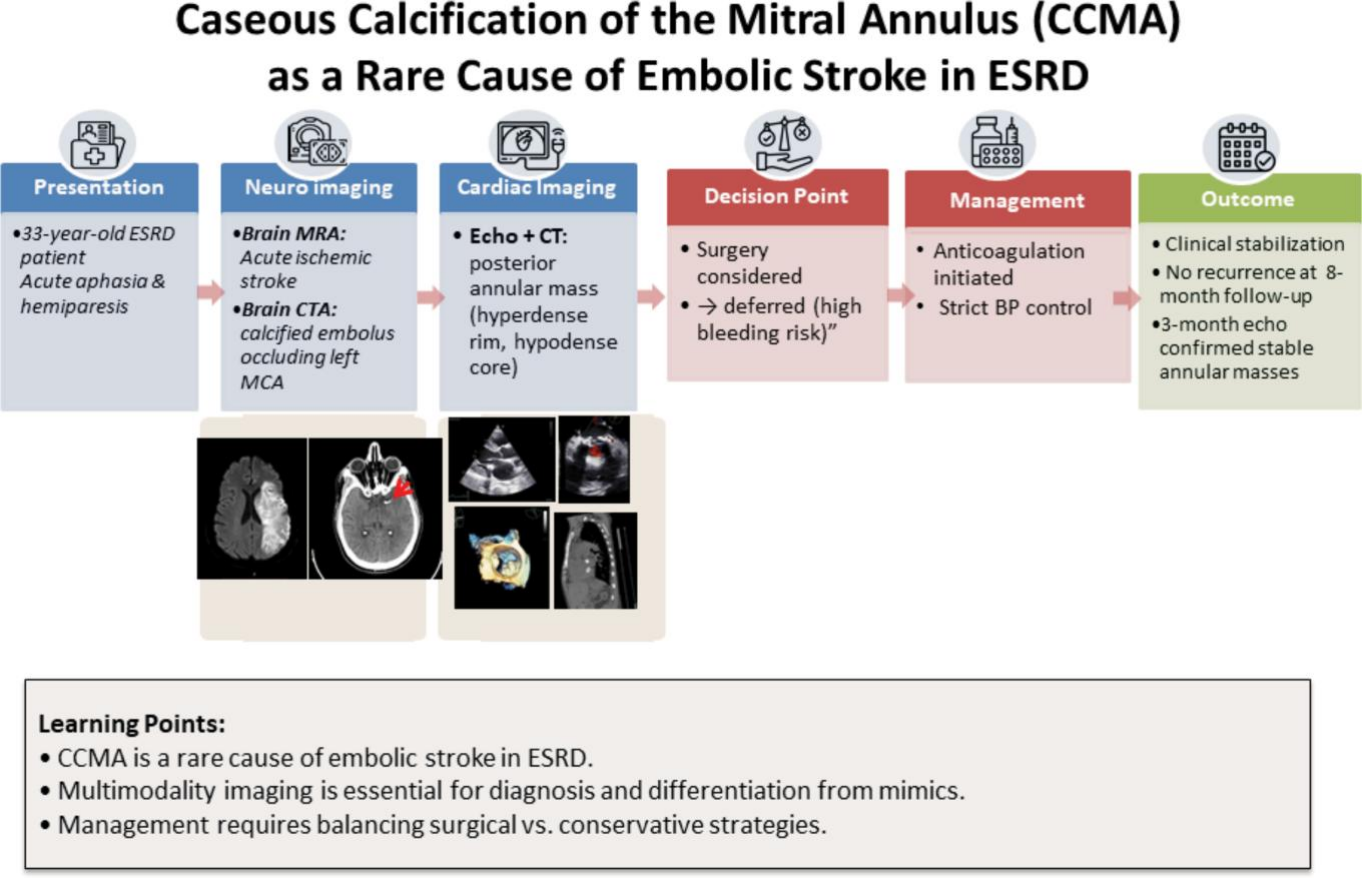
Timeline of clinical events in a 33-year-old dialysis patient presenting with acute ischaemic stroke. Brain MRA demonstrated infarction, and CTA revealed a calcified embolus in the MCA. Echocardiography and cardiac CT confirmed caseous calcification of the mitral annulus as the embolic source. A multidisciplinary team deferred surgery and opted for delayed anticoagulation with strict blood pressure control.

A 33-year-old man with ESRD secondary to long-standing hypertension had been on regular haemodialysis for three years. His hypertension was now controlled on amlodipine. He had no history of diabetes, atrial fibrillation, or prior stroke. He presented within three hours of sudden right-sided weakness and aphasia (*Figure 1*). His vital signs were within normal limits except for severe hypertension at 200/124 mmHg; he was afebrile and in sinus rhythm. Neurological examination showed complete right hemiplegia with lower facial involvement and global aphasia, without sensory or visual field deficits, giving a National Institutes of Health Stroke Scale (NIHSS) score of 21.

**Figure 2:**
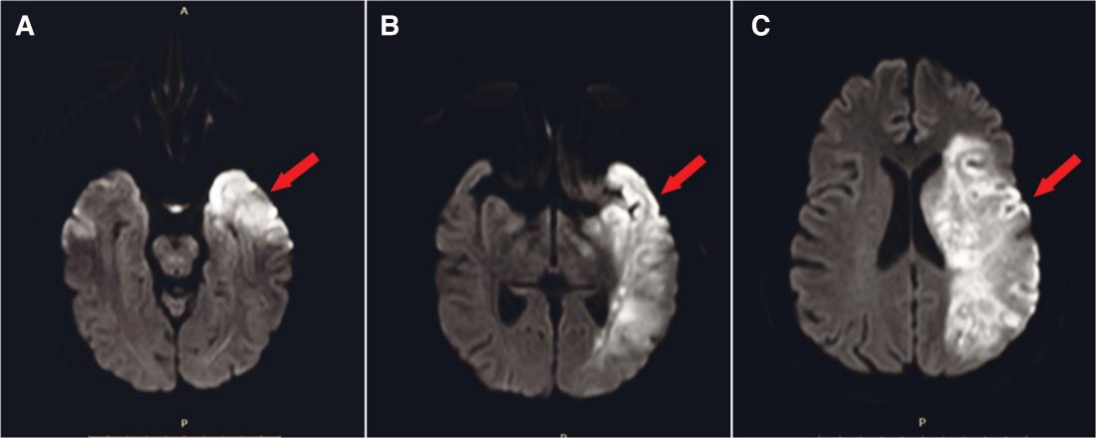
Brain MRI findings. Diffusion-weighted magnetic resonance imaging of the brain revealed increase in signal intensity in the left temporal (*A*, *B*), and parietal lobes (*C*), consistent with an acute infarct in the territory of the left middle cerebral artery. Arrows indicate the affected regions.

**Figure 3:**
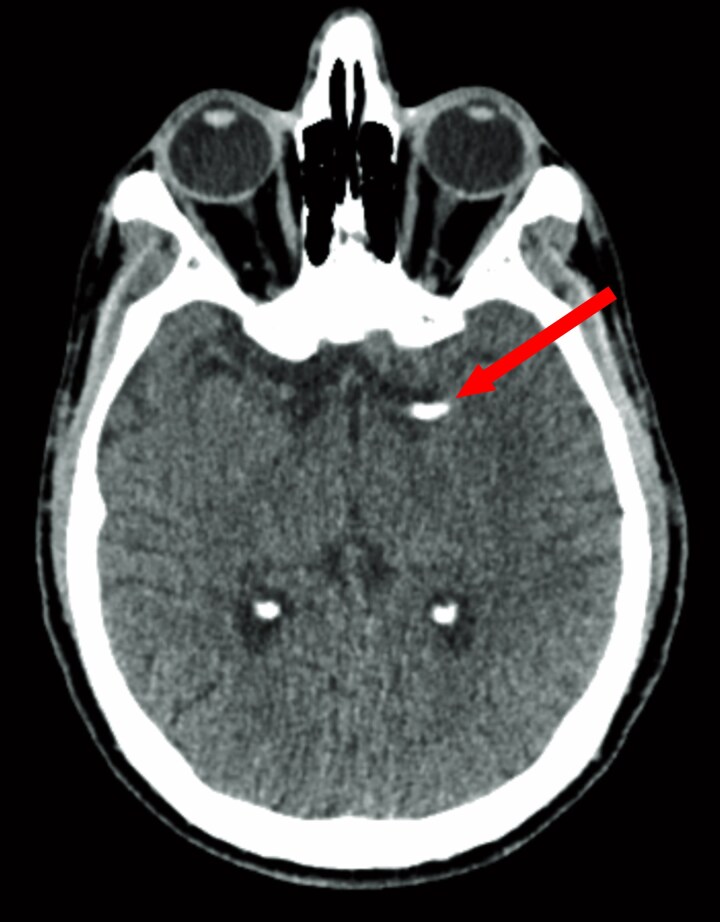
Brain CT findings. Non-contrast CT demonstrated a hyperdense intraluminal focus in the proximal M1 segment of the left MCA (arrows), measuring ∼850 HU compared with 42 HU in the contralateral M1 and ∼1350 HU in the adjacent skull table. Multi-planar reconstructions confirmed the focus centred within the lumen. CT angiography co-localized this hyperdensity with an intraluminal filling defect, consistent with a calcified embolus.

He was admitted to the stroke unit, where blood pressure was lowered gradually with intravenous nicardipine, targeting values below 180/105 mmHg. Brain MRI was obtained first because a stroke mimic was considered in this young patient.^6^ It demonstrated restricted diffusion in the left frontal and parietal regions confirmed an acute ischaemic stroke in the middle cerebral artery (MCA) territory, with a large infarct core (*Figure 2*). Brain CT angiography performed immediately afterward showed a calcified intraluminal focus occluding the proximal left M1 segment, measuring ∼850 Hounsfield units on non-contrast CT (*Figure 3*). Cervical CT angiography excluded carotid stenosis or dissection and aortic arch atheroma.

Although he arrived within the thrombolysis window, intravenous thrombolysis was withheld due to the large established core and high-risk of haemorrhagic transformation. The dense calcification of the embolus suggested a low likelihood of successful mechanical retrieval, and mechanical thrombectomy was not available at our centre.^7^ The patient was therefore managed conservatively.

Laboratory testing showed hyperkalaemia unresponsive to initial treatment, which required urgent haemodialysis without heparin. Serum calcium was 2.14 mmol/L and phosphate 2.2 mmol/L. Parathyroid hormone measured later was 380 pg/mL, and 25-OH vitamin D was 12 ng/mL, findings in line with secondary hyperparathyroidism in chronic kidney disease. Once intracranial hemorrhage was excluded, aspirin 100 mg and atorvastatin 80 mg were started, along with a phosphate binder and calcium supplementation.

**Figure 4:**
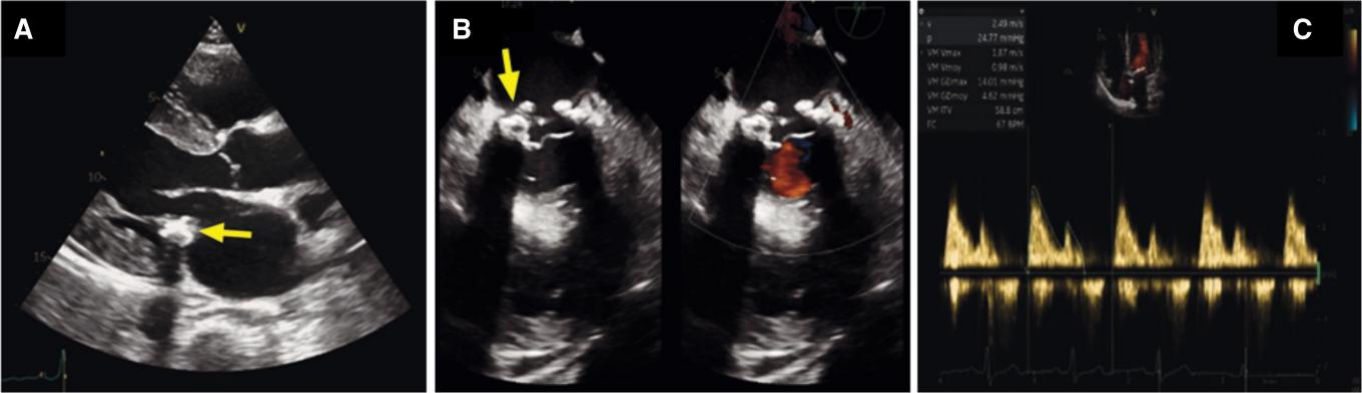
Transthoracic echocardiography findings. Parasternal long axis view (*A*) and inter commissural view (*B*), showing respectively ovoid echogenic mass of heterogeneous texture with central echolucencies (arrows) suggestive, with only mild acoustic shadowing, indicative of caseous calcification of the mitral annulus (*C*) Spectral Doppler across the mitral valve demonstrating no significant dysfunction with a mean gradient of ∼5 mmHg at a heart rate of ∼70 bpm and only mild regurgitation.

**Figure 5:**
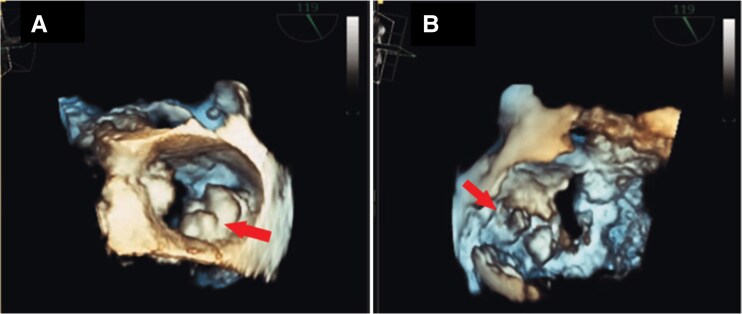
Transoesophageal echocardiography findings. 3D multi-planar reconstruction of the mitral: An En face view valve (*A*) and a ventricular view (*B*) showing extensive round to oval well delineated lobulated mass (arrows) extended along the posterior annulus, centred on the P1−P3 scallops with extension toward the posteromedial commissure and occupying the 3–5 and 7–9 o’clock positions.

**Figure 6:**
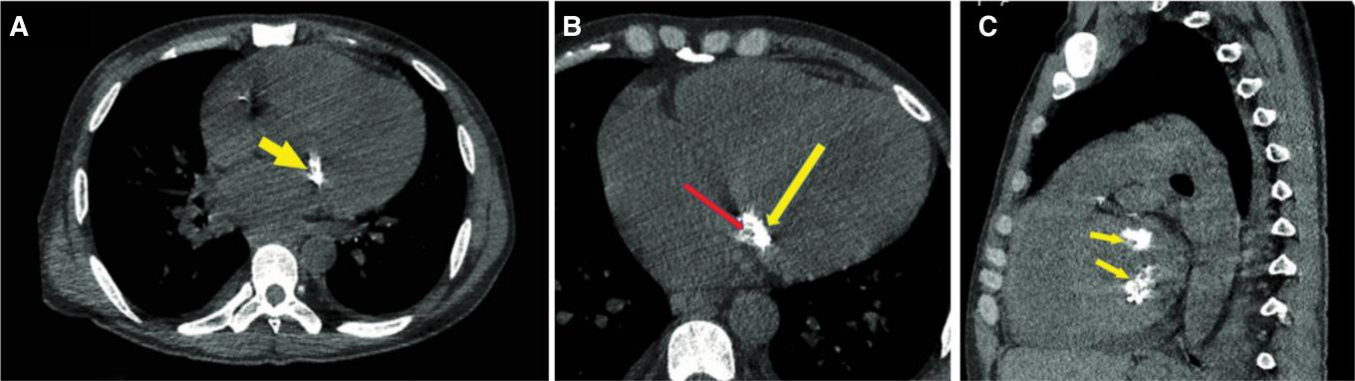
Cardiac CT findings. Axial (*A*), magnified axial (*B*), and sagittal (*C*) non-contrast ECG-gated cardiac CT views showing two lobulated posterior annular lesions with a peripheral hyperdense calcified rim (arrow; ≈880–1200 HU) and a central low-attenuation core (red arrow; ≈30 HU).

Cardiac evaluation sought a potential embolic source. Electrocardiogram and 24-h Holter monitoring showed normal sinus rhythm. Transthoracic echocardiography identified two calcified masses along the posterior mitral annulus with peripheral calcification and central echolucency. Doppler evaluation demonstrated mild mitral stenosis (mean gradient 5 mmHg) and mild regurgitation (*Figure 4*). Three-dimensional transoesophageal echocardiography localized them to the 3–5 and 7–9 o’clock positions, showing smooth lobulated contours (*Figure 5*). The contrast bubble study showed no patent foramen ovale. ECG-gated non-contrast cardiac CT confirmed two annular lesions with hyperdense rims (880–1000 HU) and hypodense cores (∼30 HU), characteristic of caseous MAC^8^ (*Figure 6*).

After multidisciplinary discussion, the lesions were considered the most plausible embolic source, consistent with prior descriptions of CCMA-related embolization.^4^ Surgical intervention was deferred due to the high peri-operative haemorrhagic risk in the context of a large recent infarct, severe hypertension, ESRD, and ongoing antiplatelet therapy reflected by a HAS-BLED score of 4. Two weeks later, after stable repeat imaging, anticoagulation with acenocoumarol (target INR 2.0–2.5) was started and aspirin discontinued, no heparin bridging was used, and blood pressure control (<140/90 mmHg) was achieved with amlodipine.

At eight-month follow-up, the patient remained free of recurrent ischaemic or embolic events. After intensive rehabilitation, his aphasia had resolved, and he showed partial motor recovery, though with significant residual neurological impairment. Once the patient had recovered from the acute phase, a surgical intervention on the mitral annulus was proposed but ultimately declined.

## Discussion

Published reports provide important context for this case. Embolic and recurrent strokes linked to CCMA have been described in elderly individuals and in patients receiving dialysis.^3,4,9^ Tan and colleagues reported a 40-year-old dialysis patient with CCMA-related stroke.^10^ The present case involves an even younger patient, in whom long-standing hypertension that progressed to ESRD, together with chronic dialysis exposure, likely contributed to the early and marked development of annular calcification.

A previously published case described massive left atrial and annular calcification with a calcified embolus in the MCA.^5^ In this patient, the radiological correspondence between the attenuation of the mitral annular lesion and the intracranial calcified embolus was stronger, although still not definitive. This concordance strengthens the plausibility of a cardiac origin while acknowledging that the link remains presumptive.

Diagnosis of CCMA can be challenging because its appearance may mimic other cardiac masses. Echocardiography usually shows a circumscribed annular mass with central echolucency and less posterior shadowing than dense MAC. Cardiac CT demonstrates a calcified rim with a hypodense core,^11^ while CMR, which is considered the gold standard, reveals a non-enhancing necrotic centre with peripheral ring enhancement.^12^ In this patient, the posterior annular location, central echolucency, and CT hypodense core within a calcified rim strongly supported the diagnosis.

Specific morphologic and functional features of MAC associated with stroke include lesion mobility, caseous necrosis, and functional mitral stenosis. These variables form the basis of a predictive MAC score used to refine cerebrovascular risk assessment.^3^ In this patient, mobility and significant stenosis were absent, but CT features were consistent with caseous necrosis, a recognized high-risk characteristic.

Several mechanisms have been proposed to explain embolization from CCMA, including surface ulceration with thrombus formation, leakage of caseous material, or extrusion of calcified fragments.^13–15^ The dense intraluminal M1 focus on cerebral CT is most consistent with extrusion of a calcified particle.

Therapeutic decision-making remains individualized because formal guideline directives for CCMA are limited. Conservative management with anticoagulation is generally preferred in the absence of significant valve dysfunction or recurrent embolic events. Habib and Rizk described a similar mass treated conservatively after detailed imaging.^8^ Surgery is considered for severe mitral stenosis or regurgitation, recurrent embolization, or uncertainty about the diagnosis.^16^ Both mitral valve repair and replacement have been reported, but replacement is often favoured because extensive debridement may extend into adjacent structures, increasing the risk of ventricular perforation and releasing necrotic material into the circulation.^17^ These concerns favour a cautious operative strategy, especially given the potential for peri-operative stroke.^4^ In this patient, surgery was deferred because of the high peri-operative haemorrhagic risk related essentially to the large recent cerebral infarction. No intervention was performed during follow-up, initially for safety reasons and later because the patient declined surgery.

This case is notable for the patient’s young age, dialysis status, and the intracranial CT showing a dense calcified MCA embolus with attenuation similar to the mitral annular lesion, supporting CCMA as a plausible source despite the absence of definitive proof. Pathological confirmation was not available, and paroxysmal atrial fibrillation could not be fully excluded. Cardiac MRI and extended rhythm monitoring were not performed, and mechanical thrombectomy was unavailable.

Within these limits, the case adds to the limited literature on CCMA-related embolic stroke in young dialysis patients and highlights the value of multimodality imaging and individualized management.

## Data Availability

Data cannot be shared for ethical and privacy reasons. The data underlying this article include clinical records and imaging studies that contain patient information. These data cannot be shared publicly to protect patient confidentiality. Deidentified data will be shared on reasonable request to the corresponding author, subject to approval by the institutional ethics committee.

## Lead author biography

Faten Yahia, MD, FEACVI, is a cardiologist at SAHLOUL University Hospital, Tunisia, with a special interest in cardiac imaging, particularly echocardiography. She enjoys working on complex cases that require cross-disciplinary knowledge and collaboration with other specialties. Passionate about continuous learning, she is equally dedicated to teaching and mentoring young colleagues.

## Notes

**Consent:** The authors confirm that written informed consent for submission and publication of this case report, including images and associated text, has been obtained from the patient in line with COPE guidelines.
